# Supplementary material for: Ultra-thin van der Waals crystals as semiconductor quantum wells
Source: Nat Commun. 2020 Jan 8;11:125. doi: 10.1038/s41467-019-13893-w (PMC6949292; doi:10.1038/s41467-019-13893-w)
Supplement: Supplementary file 1 — Supplementary Information [file 41467_2019_13893_MOESM1_ESM.pdf]

Supplementary Information: Ultra-thin atomic films as semiconductor quantum wells

Zultak et al.

Supplementary Note 1 Fabrication

The device used for tunneling measurement was built from an InSe crystal displaying terraces, as shown on Fig. 1a,b. Each of the areas defined by the top electrodes displayed electroluminescence that could be controlled independently, with example EL map showed in Fig.1c of the main text and its representative spectra showed in Fig. 1c. For the accurate determination of the barrier thicknesses the device topography have been mapped using atomic force microscope, see Fig. 2.

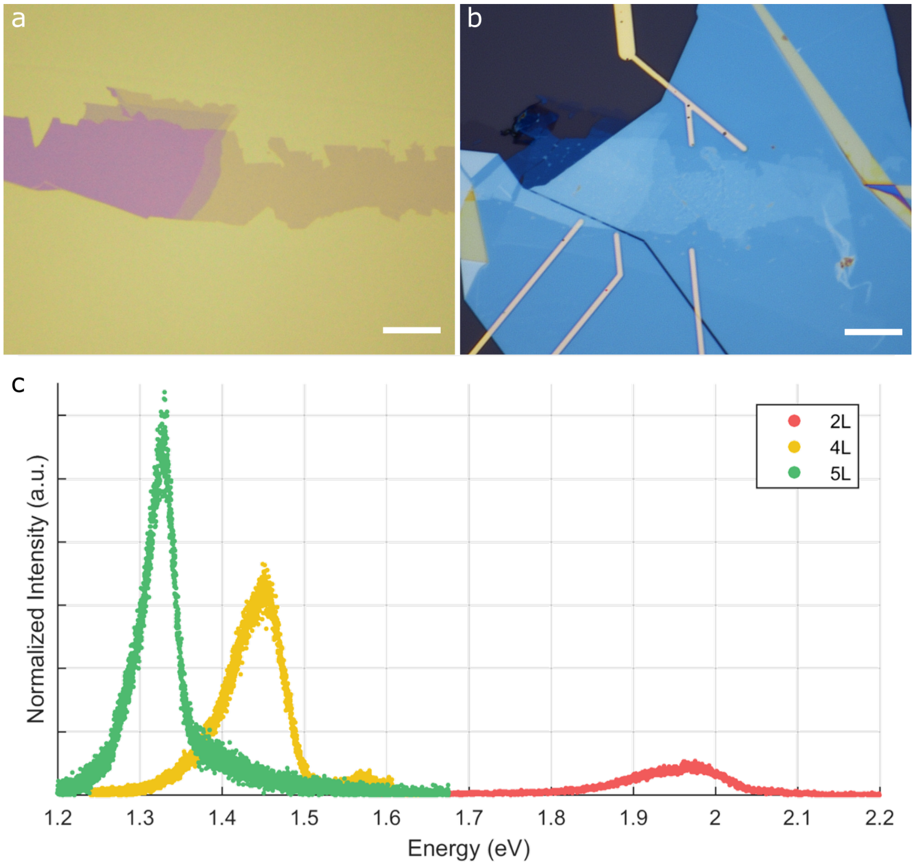

Supplementary Figure 1: **(a)** Optical micrograph of a selected InSe crystal displaying areas of 1, 2, 3, 4 and 5L thickness. **(b)** Complete LED device produced using InSe crystal shown in (a). **(c)** Representative EL spectra of the three InSe thickness (2, 4 and 5L). The scale bars are 10 µm.

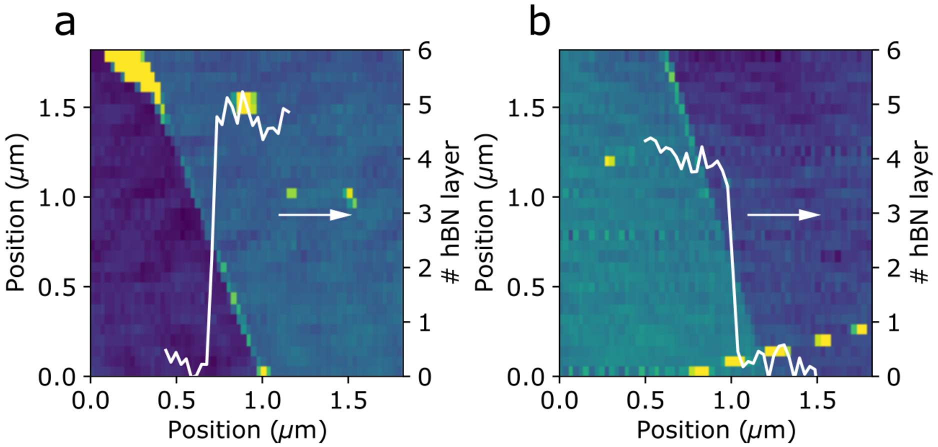

Supplementary Figure 2: Atomic force micrographs of the (a) top and (b) bottom hBN flake with extracted height profile in white for the device presented in Fig. 1 showing the asymmetrical tunnel barriers of 4 (top) and 3 (bottom) layers.

Supplementary Note 2 Measurements

Current density measurement

The current density derivative shown in Fig.2e,f of the main text were obtained by recording the current density, shown in Fig. 3, and numerically differentiating it to obtain the dI/dV_b curves.

The bias axis of Fig.2 of the main text has been converted into energy using the measured threshold for electroluminescence for each side of the bias and the previously reported value (ARPES) for the energy separation between the maximum of the valence band of InSe and the Dirac cone of graphene, $1.32\,\mathrm{eV}$ for 4 and 5 layers (Fig. 4 and reference [1]). The conversion is then given by the ratio of $1.32\,\mathrm{eV}$/(EL onset on the other side of V=0 axis). The asymmetry in the barrier thicknesses causes the variation in the onset of the electroluminescence for opposite signs of the bias. This is done independently for the positive and negative axis of the four and five layers sample.

ARPES data for band alignment

The band alignment shown in Fig2a of the main text were extracted from ARPES data presented in [1]. The measurement is presented in Fig. 4a,b,c for graphene over InSe and 5L, 7L InSe alongside a diagram (Fig. 4d) showing the band alignment extracted from the ARPES data for various thicknesses of InSe.

PLE for in plane polarized light

The PLE spectra shown on Fig. 5 for conventional in-plane sample orientation display prominent A and B transitions that have previously been reported in PL measurements [2]. Due to

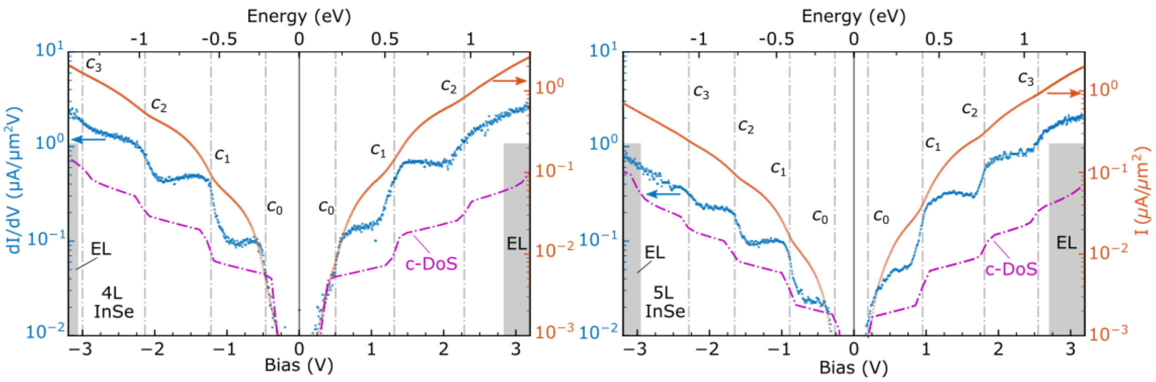

Supplementary Figure 3: $I(V_b)$ (orange), dI/dV_b (blue) and density of states of the conduction bands from tight binding model (purple, arb. u.) for 4L left and 5L right InSe film, with the number of layers established by AFM topography. The shaded areas indicated the electroluminescence region Energy scale along the top axis was found using the EL onset values and the band alignment from ARPES.

inefficient coupling of in-plane polarized light features between A and B transitions cannot be clearly identified unlike in lamellae specimen presented in the Fig3d,e of the main text.

For the determination of the exciton binding energy, the lowest energy peak has been fitted using a Gaussian, as showed in the Inset of Fig.3c in the main text, and subtracted from the PLE spectrum. The difference between the peak position and the highest derivative point of the recovered step has been taken as the binding energy while the limits of the steps give the error bars.

Cross-sectional STEM

Lamellae were prepared using focused ion beam as shown in Fig. 6. Energy dispersive x-ray spectroscopy (EDS) was performed with the Titan′s Super-X $0.7\,\mathrm{srad}$ 4-detector EDS system, a $50\,\mu\mathrm{s}$ dwell time and 19 minute total acquisition time. EDS spectrum image data was averaged along a $\sim 10\,\mathrm{nm}$ width of the heterostructure to produce the mean EDS intensity line scan data in Fig. 7.

Supplementary Note 3 Theory

Inclusion of spin-orbit coupling in tight-binding model

The theoretical calculations presented here use the tight-binding model from [3], which did not take spin-orbit coupling (SOC) into account. Its effect on the symmetry and polarization of the principal interband optical transitions are however, important[4, 5], so for calculation of the optical absorption and its dependence on polarization it must be included. Taking the Hamiltonian of [3], H_0, as a starting point, atomic SOC is added between p orbitals on the same

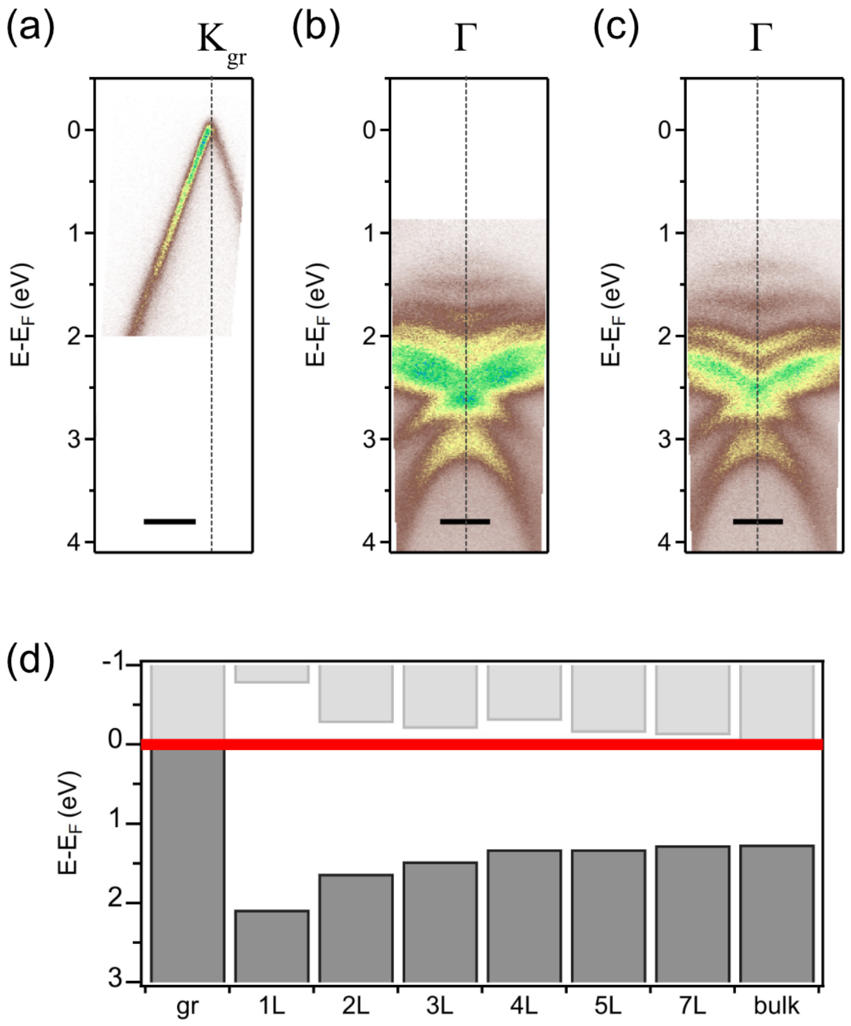

Supplementary Figure 4: Band alignments of graphene on InSe. ARPES energy-momentum slices at the corner of the graphene Brillouin zone, (a), and at the zone centre on 7L InSe, (b), and 5L InSe, (c). Within the uncertainty of the measurement, the graphene is undoped with the Dirac point at the Fermi level. The layer-dependent InSe valence band maximum can be directly determined from this data. (d) Schematic of the band energies and band alignments for graphene compared to N-layer InSe as labelled. The valence band maximum (top of the dark grey boxes) is determined from ARPES data; the conduction band minimum (bottom of the light grey boxes) is determined by adding the optical gap to the VBM, assuming a small exciton binding energy. Data taken from [1].

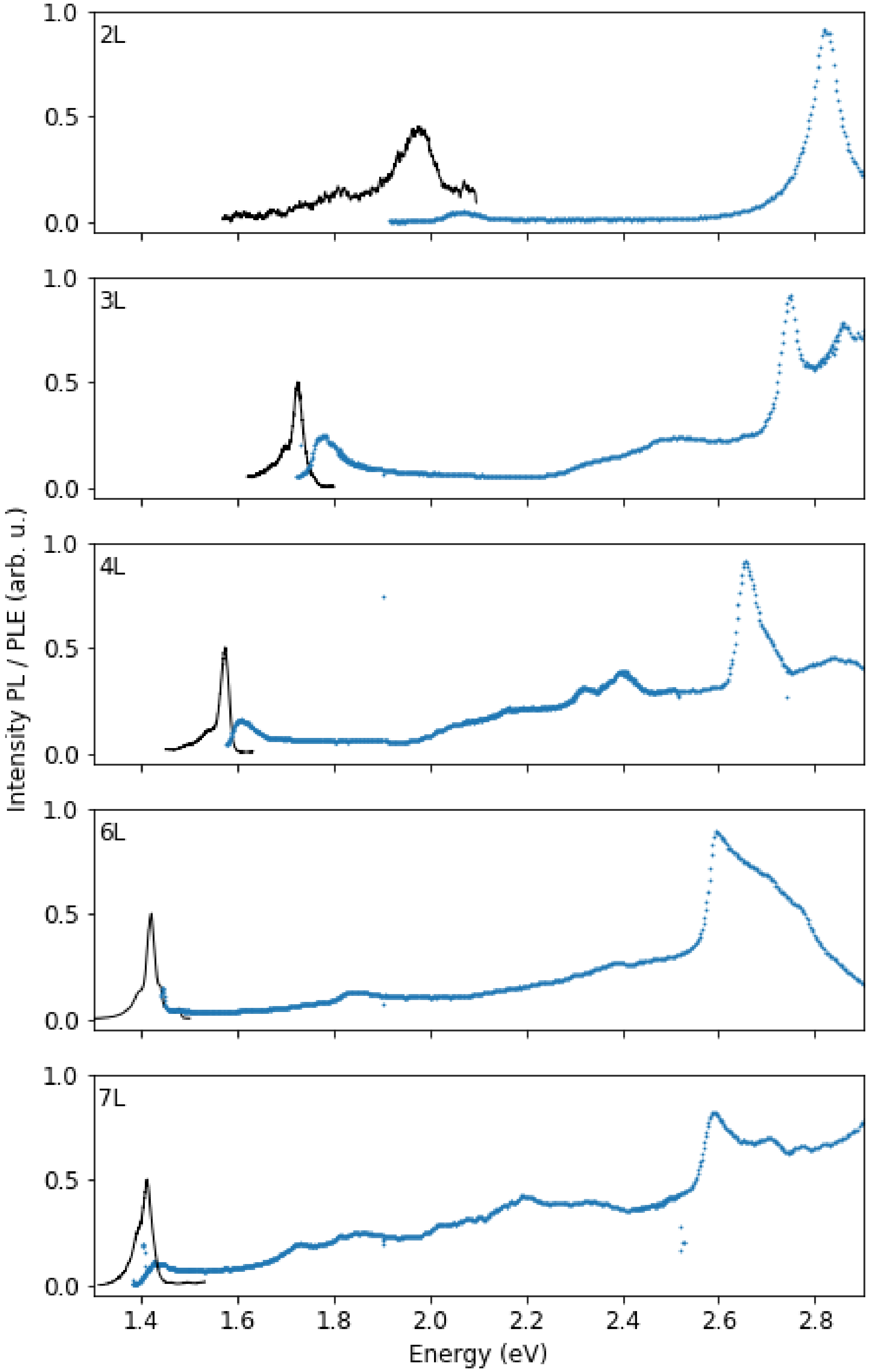

Supplementary Figure 5: PL (black) and PLE (blue) spectra for out-of-plane propagating light for various thicknesses of hBN-encapsulated InSe.

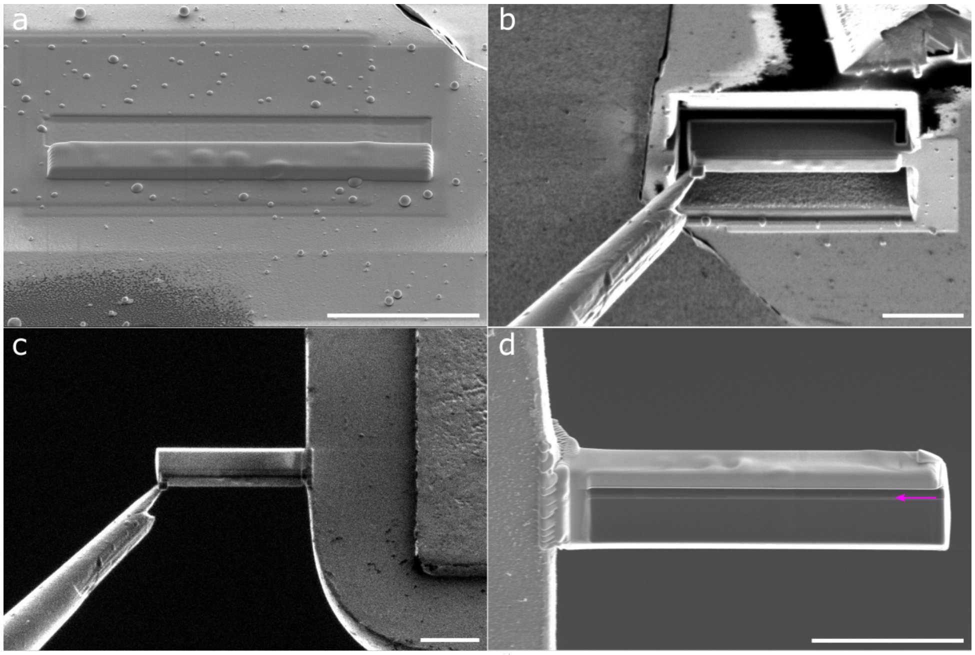

Supplementary Figure 6: Lamella extraction process. (a) Electron-beam-assisted deposition of Pt protection layer (b) Ga+ ion milling prior to lamella lift-out (c) Re-attachment of the specimen to the TEM grid using OmniProbe micromanipulator (d) The specimen after polishing procedure, the position of InSe layer is indicated by the purple arrow. The scale bars are $10\,\mu\mathrm{m}$.

atomic site as

$$
\begin{aligned}
H = H_0 \otimes \mathbb{1}_s + \sum_{j,n,f,\mu} \Big[& \lambda_{In} \left(-2i\mu m^{\mu\dagger}_{(n)fjp_x} m^{\mu}_{(n)fjp_y} + 2\mu m^{\mu\dagger}_{(n)fjp_x} m^{-\mu}_{(n)fjp_z} - i m^{\mu\dagger}_{(n)fjp_y} m^{-\mu}_{(n)fjp_z} \right) \\
& + \lambda_{Se} \left(-2i\mu x^{\mu\dagger}_{(n)fjp_x} x^{\mu}_{(n)fjp_y} + 2\mu x^{\mu\dagger}_{(n)fjp_x} x^{-\mu}_{(n)fjp_z} - i x^{\mu\dagger}_{(n)fjp_y} x^{-\mu}_{(n)fjp_z} \right) \Big] \\
& + \mathrm{H.c.}.
\end{aligned}
\tag{1}
$$

Here, the sum runs over unit cell j, layer n, sublayer $f = 1, 2$ and spin projection $\mu = \mathbf{s} \cdot \hat{\mathbf{e}}_{\mathbf{z}} = \pm\frac{1}{2}$. $\mathbb{1}_s$ is a 2×2 identity matrix in $\mu = \pm\frac{1}{2}$ spin space. The operator $m(x)^{\mu(\dagger)}_{(n)fip_\alpha}$ annihilates(creates) an electron in orbital p_α with spin projection μ on an In(Se) atom in unit cell j, layer n, sublayer f of the crystal. We set the atomic spin-orbit coupling constants, λ_{In} and λ_{Se}, as λ_{In}=$0.15\,\mathrm{eV}$, λ_{Se}=$0.15\,\mathrm{eV}$ based on the splitting of $\mathrm{In}_{p_{x,y}}$-dominated and $\mathrm{Se}_{p_{x,y}}$-dominated DFT bands on taking SOC into consideration [6].

Optical absorption

To find the single-particle optical absorption for in-plane polarized light, illustrated for 2 to 7 layers of Inse on Fig. 9, we adapt the analysis of [3] and replace the transitions between

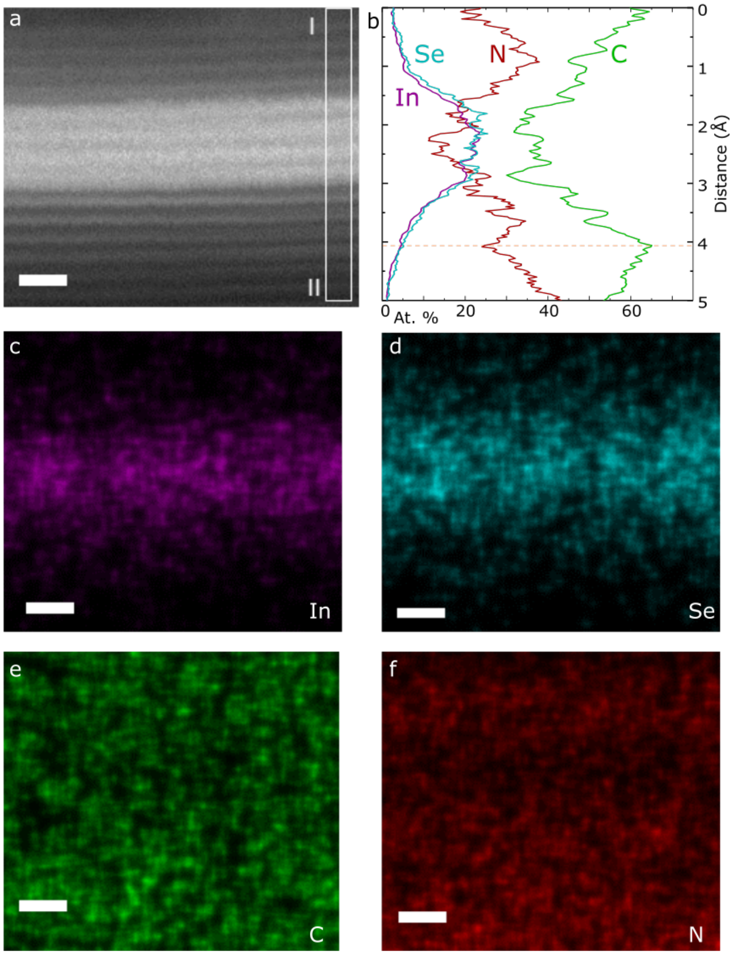

Supplementary Figure 7: EDS analysis of Gr-BN-InSe-BN-Gr. (a) High-angle annular dark-field (HAADF) image, in which the layers with the brightest contrast is from InSe, (b) the atomic-percent distribution along line I-II of (a), calculated from EDS mapping. The dotted line indicate the bottom graphene location. (c-f) Corresponding raw EDS elemental maps of (c) In, (d) Se, (e) C, (f) N. Scale bars are $2\,\mathrm{nm}$.

individual pairs of bands at Γ with a sum over all bands over the whole Brillouin zone

$$g(\hbar\omega) = \frac{8\pi^2}{A_{uc}N_{\mathbf{k}}}\frac{e^2}{\hbar c}\sum_{c,v,\mathbf{k}}\frac{|\langle c|\,\partial_x H\,|v\rangle|^2}{\hbar\omega}\delta(\hbar\omega - E_{cv\mathbf{k}}), \tag{2}$$

where A_{uc}=$13.53\,\mathrm{\AA}^2$ is the area of a unit cell, $N_{\mathbf{k}}$ is the number of $\mathbf{k}$-points in the sum over the Brillouin zone, and $\frac{e^2}{\hbar c}$ is the fine structure constant. The sums over v and c run over all occupied and unoccupied bands, respectively. The delta function $\delta(\hbar\omega - E_{cv\mathbf{k}})$ between the photon energy $\hbar\omega$ and the energy difference between bands c and v at $\mathbf{k}$, $E_{cv\mathbf{k}}$, is replaced by a Gaussian broadening of width $5\,\mathrm{meV}$ in this work. In the main text only the lowest unoccupied subband c_0 is included in the sum.

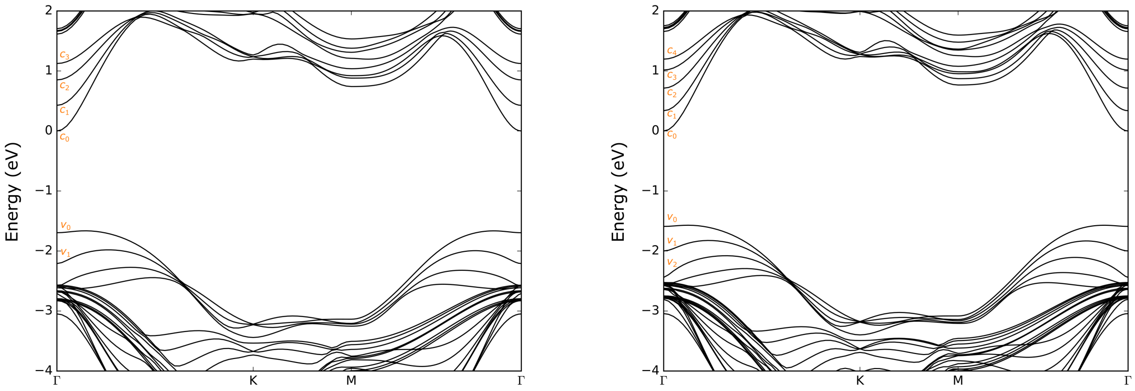

Supplementary Figure 8: Band Structure of InSe

For out-of-plane polarization, the expression is modified to read

$$g(\hbar\omega) = \frac{8\pi^2}{A_{uc}N_{\mathbf{k}}}\frac{e^2}{\hbar c}\hbar\omega\sum_{c,v,\mathbf{k}} |\left\langle c\right| z \left|v\right\rangle|^2\delta(\hbar\omega - E_{cv\mathbf{k}}), \tag{3}$$

where $\left\langle c\right| z \left|v\right\rangle$ is the out-of-plane position matrix element between bands c and v (measured about the mean plane of the crystal).

Density of states for tunneling current computations

To compute the bias-dependent density of states, at each bias for which we calculate the DoS we use Eq. (main text 1) to determine the electric field across the InSe, then determine the energy, E, at which we calculate the DoS using the linear relations shown by the dashed lines in Fig.2(g,h) of the main text. The bias-dependent DoS is then computed as

$$\mathrm{DoS}(E) = \frac{1}{A_{uc}N_{\mathbf{k}}}\sum_{i,\mathbf{k}}\delta(E - E_{i,\mathbf{k}}) \tag{4}$$

where the sum is over bands i, momentum $\mathbf{k}$ which have energy $E_{i,\mathbf{k}}$. $N_{\mathbf{k}}$ is the number of $\mathbf{k}$ points in the sum, and A_{uc}=$13.53\,\mathring{\mathrm{A}}^2$ is the 2D unit cell area. We approximate the delta function by a Gaussian broadening of $20\,\mathrm{meV}$. Since the sharp increase in the DoS on reaching the bands around the M point is not evident in the tunnelling behaviour we leave out the $\mathbf{k}$ points around M from the sum - since the splitting in energy between the conduction band minima at Γ and M is sensitive to the choice of lattice parameter/DFT functional [6] such a discrepancy between the tight-binding model (which was originally parametrised to DFT) and experiment is not necessarily surprising.

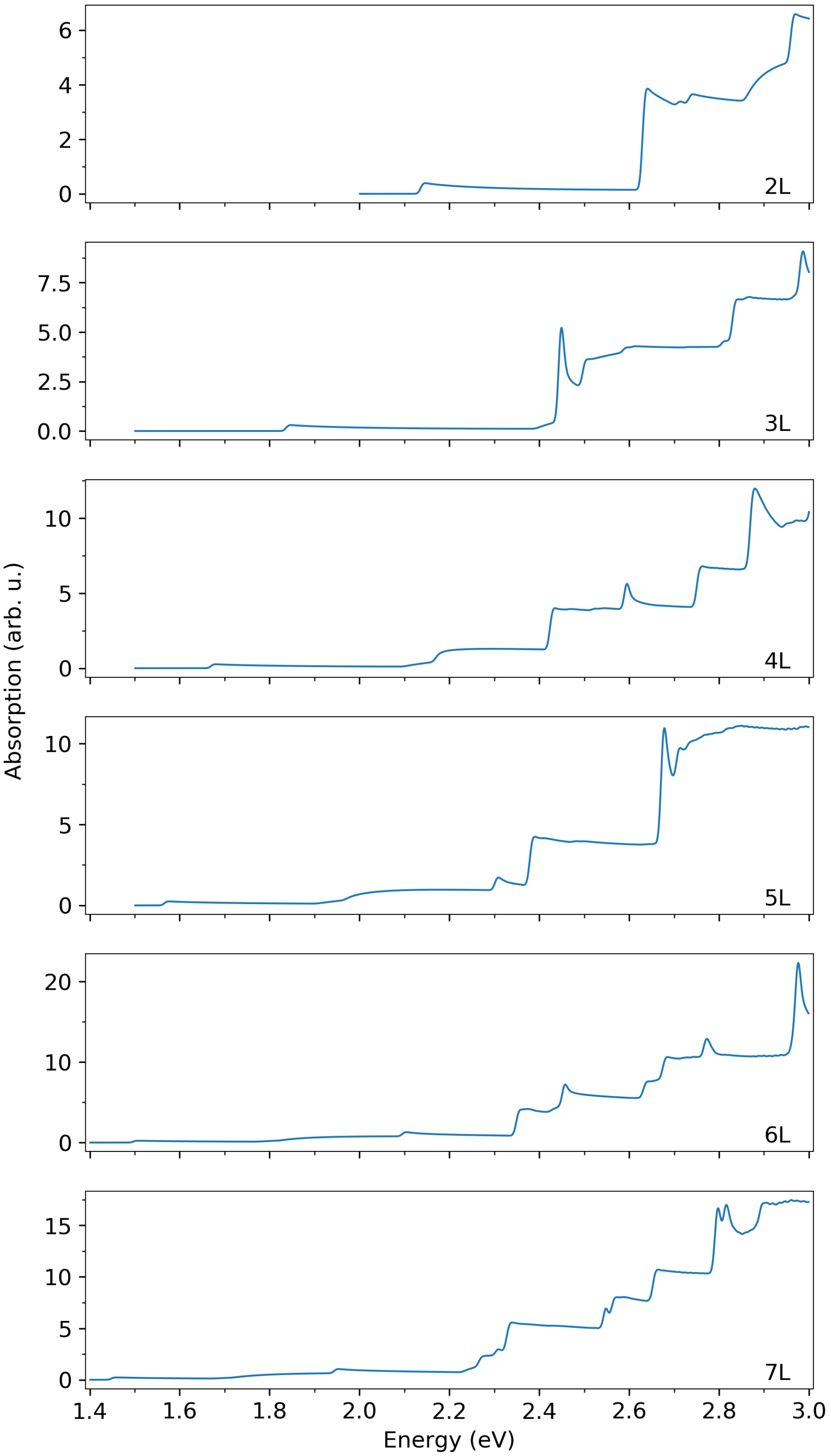

Supplementary Figure 9: Optical absorption for 2 to 7 layer of InSe.

Supplementary References

1. Hamer, M. J. *et al.* Indirect to Direct Gap Crossover in Two-Dimensional InSe Revealed by Angle-Resolved Photoemission Spectroscopy. *ACS Nano,* 2136–2142 (2019).
2. Bandurin, D. A. *et al.* High electron mobility, quantum Hall effect and anomalous optical response in atomically thin InSe. *Nature Nanotechnology* **12,** 223–227 (2017).
3. Magorrian, S. J., Zólyomi, V. & Fal'ko, V. I. Electronic and optical properties of two-dimensional InSe from a DFT-parametrized tight-binding model. *Physical Review B* **94,** 245431 (2016).
4. Magorrian, S. J., Zólyomi, V. & Fal'ko, V. I. Erratum: Electronic and optical properties of two-dimensional InSe from a DFT-parametrized tight-binding model [Phys. Rev. B 94 , 245431 (2016)]. *Physical Review B* **96,** 079905 (2017).
5. Magorrian, S. J., Zólyomi, V. & Fal'ko, V. I. Spin-orbit coupling, optical transitions, and spin pumping in monolayer and few-layer InSe. *Physical Review B* **96,** 195428 (2017).
6. Zólyomi, V., Drummond, N. D. & Fal'ko, V. I. Electrons and phonons in single layers of hexagonal indium chalcogenides from ab initio calculations. *Physical Review B* **89,** 205416 (2014).
